# Patient directed social media use among participants of centering pregnancy groups

**DOI:** 10.1186/s41043-025-01159-9

**Published:** 2025-12-29

**Authors:** Alison M. El Ayadi, Nadia G. Diamond-Smith, Mia Schuman, Laura Weil

**Affiliations:** 1 Department of Obstetrics, Gynecology and Reproductive Sciences, University of California San Francisco, San Francisco, CA USA; 2 Department of Epidemiology and Biostatistics, University of California San Francisco, San Francisco, CA USA

**Keywords:** Group prenatal care/Centering Pregnancy^®^, Antepartum care, Postpartum care, Health informatics, Mental health, Patient education

## Abstract

**Introduction:**

Social support in pregnancy and postpartum is important for optimizing maternal and infant health. Group prenatal care offers the opportunity for in-person social support yet does not extend into the postpartum period. Mobile social support models may further meet the needs of pregnant individuals and partners during pregnancy and into the postpartum period. However, the use and utility of mobile social support for pregnant people and their partners in the context of group prenatal care and beyond has not been studied. Assessing Centering patients’ utilization of existing social media platforms can inform programmatic development.

**Methods:**

We conducted a retrospective cross-sectional study among recent participants of UCSF’s Centering Pregnancy^®^ program and their partners. Study participants were recruited through UCSF’s electronic health record system or direct email and partners were recruited through referral from participants. Online surveys sought to understand participant perspectives on mobile groups, educational and social support needs, and recommendations.

**Results:**

Participants gave birth between 2018 and 2021 (68%), were college-educated (97.3%), and regularly accessed social media (> 75% across platforms). Most participants engaged in Centering Pregnancy^®^ online communications outside of formal activities (79% during pregnancy, 74% postpartum) for social support (78.1%) and knowledge sharing (65.2%). Most posted content monthly (54.6%) but read content more frequently (48.0% at least weekly). Communication frequency and topics changed with the COVID-19 pandemic. Respondents wanted more information on infant sleep (42.6%), maternal recovery/health (38.7%), breastfeeding/formula feeding (37.4%), newborn health and care (34.2%), and child development (27.1%). Social group engagement was higher for individuals reporting depressive or anxiety symptoms during pregnancy and postpartum.

**Discussion:**

The findings confirm the importance of social support, especially postpartum, for health, how mobile support groups can impact these outcomes, and needs and areas of improvement for group prenatal care. Integration of a social media support component to the evolving post-COVID Centering Pregnancy^®^ model may be an important addition to improve participating parent wellbeing.

## Introduction

Social support during pregnancy and postpartum is strongly associated with improved maternal physical and mental health, as well as better birth outcomes, particularly in high-risk populations [1–3]. Despite its demonstrated importance, perinatal social support interventions remain underutilized in current mainstream care models, particularly during the postpartum period. Research has shown that women with low self-reported social support are at higher risk of depressive symptoms, reduced quality of life, and in certain subgroups, adverse birth outcomes such as preterm birth or low birth weight infants [2, 4, 5]. These findings underscore the urgent need for innovative, low cost approaches to ensure that pregnant and postpartum individuals receive the emotional, instrumental, informational, and appraisal support they need during a stressful and often isolating time [6]. 

Social media use during pregnancy and postpartum has emerged as a promising avenue for enhancing emotional and informational support, especially in the wake of the COVID-19 pandemic, which shifted possible aspects of healthcare delivery to virtual formats. Studies estimate a majority of recently pregnant patients use social media for information and advice about pregnancy and parenting (89%). [7–9] One study found that 83% of respondents identified *“friends from social media”* as key sources of social support for parenting [7]. Numerous social media interventions have been developed to address the support needs of pregnant and postpartum individuals. A recent meta-analysis concluded that social media interventions can effectively promote healthy behaviors related to gestational diabetes, weight management, and maternal mental health [10]. Similarly, a scoping review of perinatal health promotion interventions using social media demonstrated significant improvements in breastfeeding practices and parenting confidence [11]. These findings from both observational and interventional studies highlight the potential of social media platforms to provide meaningful social support, particularly when structured around small group interactions.

Fostering social support is an explicit focus of group prenatal care models such as Centering Pregnancy^®^, women’s self-help groups, and other similar initiatives. These models deliver prenatal care in a group setting, integrating clinical assessment with education, discussion, and social interaction. The primary aim of group prenatal care is to provide efficient, equitable care while promoting improved birth outcomes through increased patient-provider engagement, patient empowerment, and peer support among participants. Each session includes facilitated educational activities and group discussions. Partners are explicitly included in this model to enhance their engagement, education, and support for the birthing individual. Typically, group sessions occur during the second and third trimesters of pregnancy, with some programs offering a final postpartum session. However, most group prenatal care models do not extend formally into the postpartum period.

Group prenatal care has gaining popularity in the U.S. due to high acceptability and demonstrated positive outcomes, including reductions in preterm births, caesarean surgeries, and low birth weight rates;, improved maternal weight gain; increased exclusive breastfeeding; higher postnatal contraceptive uptake and infant vaccination rates and reduced postnatal depression [12–22]. Women from historically minoritized racial or ethnic groups and underserved communities have been found to experience the most significant benefits from group prenatal care interventions [23]. 

The University of California San Francisco (UCSF) Women’s Health Center has been a leader in expanding access to group prenatal care. One recent innovation involves encouraging group prenatal care participants to form mobile social support groups to foster interaction beyond the formal, provider-facilitated meetings and extending into the postpartum period after group prenatal care concludes. Anecdotal evidence suggests that some groups have already begun communicating via platforms such as WhatsApp and other forums. However, no known studies have examined the feasibility, acceptability, and impact of these mobile social support groups on overall social support, nor have they explored the primary content areas shared within these groups among Centering Pregnancy participants.

Understanding the role of mobile social support as a complement to group prenatal care is vital for continuing to refine this care model. Identifying the types of support and information that pregnant individuals and their partners seek outside of group care can help healthcare providers deliver more targeted and effective support throughout pregnancy and postpartum and contribute to expanding the evidence base on innovative approaches to maintain high quality care through virtual telehealth models which gained heightened attention during the recent COVID-19 pandemic which necessitated shifts in care delivery to virtual platforms.

To address these gaps in research and practice, we aimed to explore the use of social media as adjunct to the group care model among UCSF’s Centering Pregnancy^®^ participants both during and after pregnancy. We focused on understanding group formation, participation and dynamics, main topics of discussion, perceived benefits and drawbacks to social media use as adjunct, and its potential correlations with mental health symptoms among birthing individuals and their partners.

## Methods

### Study overview

We conducted a retrospective cross-sectional study among recent participants of UCSF’s Centering Pregnancy^®^ program, which has been a cornerstone of prenatal care at UCSF since its inception in 1999. Each year, approximately 150–200 women participate in Centering Pregnancy^®^, with 1–2 groups launched monthly. These groups are led by a faculty midwife or physician, often with involvement from obstetrics and gynecology residents. Participation is opt-in and the program is in considerable demand, with a waitlist for enrollment. Patients are typically enrolled following their initial obstetrics intake appointment and include both low and high-risk patients, some of whom are co-managed by high-risk obstetricians. UCSF serves a unique patient population reflective of a large academic medical center in a major U.S. city, with participants tending to be highly educated, higher income, and disproportionately white [24]. 

## Participant recruitment

Potential participants were identified and recruited via UCSF’s electronic health record (EHR) system and via direct program email. Eligibility criteria included having attended at least one group prenatal care visit in the prior five years and giving birth to a live infant. Recruitment emails were sent through the patient-provider messaging system and included a summary of the research aims, a description of survey participation, details on confidentiality measures, and potential risks and benefits. Individuals who responded that they were interested in learning more about the study were connected to the research team who shared further details regarding the study procedures and a link for online participation. Participants confirmed informed consent by checking a box before being allowed to access the survey. To maintain participant confidentiality, identifying information such as names was not collected for research purposes. However, email addresses were separately collected solely for distributing a participation incentive of a $10 Amazon.com gift card. Recruitment emails also included a link inviting participants to share the study with their partners, who were eligible to complete a similar online survey.

## Study measures

The participant survey captured sociodemographic characteristics (e.g., age, parity, educational attainment, race/ethnicity, and general social media use) to describe our sample. Additional questions focused on sources of social support (technological, in-person, etc.), frequency of engagement, and changes in social support engagement across the pregnancy and postpartum continuum. Specific questions addressed social media group participation, including the mobile platforms used, participants’ perspectives on the benefits and challenges of the social media group, perceived value, impact on social support, and suggestions for improvement. Participants were also asked to assess how well their emotional and informational social support needs were met during pregnancy and postpartum. Mental health, specifically depressive and anxiety symptoms, was evaluated using a one-item likert-type scale for each domain reflecting symptom frequency, adopted from the Pregnancy Risk Assessment Monitoring System (PRAMS) [25]. Participants were also asked whether they had received a clinical diagnosis of depression and anxiety. Finally, participants were asked to share what additional information or support they would have liked during pregnancy and postpartum, as well as recommendations for creating a more formal social media group within the context of UCSF’s Centering Pregnancy^®^ program.

Recruitment occurred in two phases. During the initial phase, between October 22 – December 31, 2020, 731 individuals were identified as eligible for participation and sent the initial recruitment email within UCSF’s EHR messaging system. A total of 106 expressed interest in the study, and 81 completed the online survey. Partners were invited during this phase via link sharing from participants, with 12 partners completing the online survey. A second round of recruitment was conducted between July 15 – August 16, 2022, targeting all 234 pregnant individuals who participated in UCSF’s virtual Centering Pregnancy^®^ program, a COVID-19 pandemic adaptation of the traditional, in-person model, including patients who were currently pregnant and actively receiving group care. Virtual groups were offered from 2020 to 2023, after which a hybrid model was implemented. Among the second sample, 74 individuals completed the survey, with demographic characteristics similar to those of the first round and reflective of UCSF’s broader larger Centering Pregnancy^®^ population. Partner recruitment followed the same strategy, though partner participation remained limited.

### Data analysis

Data analysis focused primarily on responses from birthing individuals, although partner data were included where available, though the sample size is considerably smaller. Analyses were largely descriptive, with missing data noted. Socio-demographic characteristics, engagement in adjunct social media groups, and their emotional and informational support and needs were summarized using frequencies and proportions. To explore the relationship between social media group engagement and mental health symptoms, we used logistic regression analysis to estimate the association between n frequency of social media group engagement (e.g., reading and posting) and self-reported depression and anxiety. Mental health symptoms were operationalized as “always” or “often” versus “sometimes”, “rarely”, or “never”. Differences in depression and anxiety between pregnancy and postpartum were assessed using Stuart-Maxwell’s test.

## Ethics

All study procedures and survey instruments were reviewed and approved by the UCSF Human Research Protection Program IRB#:20–30838. All participants provided online confirmation of informed consent prior to completing the survey. This research article was written in accordance with the STROBE guidelines for cross-sectional studies.

## Results

### Survey participant characteristics

A total of 155 study participants complete the survey, ranging in age from 27 to 46, with most between age 35–39 (49.7%; Table 1). Most participants had given birth between 2018 and 2021 (67.7%) and were experiencing their first pregnancy (63.9%). Nearly all participants were college-educated (97.3%), with 65.8% holding a graduate degree. The majority identified as white (60.0%). Participants were well-versed in online communication, with most regularly accessing social media and electronic communication platforms weekly, including Facebook or Instagram (75.5%), email (74.2%), and WhatsApp or other text applications (74.2%). Engagement in Centering Pregnancy^®^ in-person meetings was high, with 93.5% reporting having participated in all or most of the group meetings (not shown).

**Table 1:** Sociodemographic characteristics of UCSF centering Pregnancy^®^ Participants and partners engaging in social media survey

	Participant (*n* = 155)		Partner (*n* = 22)	
	*n*	%	*n*	%
Age Group				
< 30	5	3.2	0	0.0
30–34	51	32.9	5	23.8
35–39	77	49.7	13	61.9
40+	22	14.2	3	14.3
Year Gave Birth				
2016	9	5.8	1	4.8
2017	10	6.5	2	9.5
2018	29	18.7	1	4.8
2019	22	14.2	2	9.5
2020	20	12.9	7	33.3
2021	34	21.9	4	19.0
2022	23	14.8	2	9.5
Haven’t given birth	8	5.2	2	9.5
Number of times pregnant	(*n* = 153)			
1	99	64.7		
2–3	51	33.3		
4 or more	3	2.0		
Pregnancy participated in Group ANC				
First	99	63.9		
Second	56	36.1		
Educational attainment				
Some college	4	2.6	1	4.8
Bachelor’s degree	49	31.6	9	42.9
Graduate degree	102	65.8	11	52.4
Race/ethnicity	(*n* = 150)			
White	90	60.0	10	47.6
Asian	32	21.3	8	38.1
Multiple	13	8.7	1	4.8
Hispanic	9	6.0	1	4.8
Black	3	2.0	0	0.0
Other	3	2.0	1	4.8
Social media platforms used on weekly basis				
Emails	115	74.2	17	77.3
Facebook or instagram	117	75.5	10	45.5
Slack	51	32.9	10	45.5
Zoom or skype	73	47.1	12	54.5
WhatsApp or other texting	115	74.2	18	81.8
Other	8	5.2	3	13.6

Twenty-two partners also completed the survey (Table 1). Most partners were aged 35–39 (61.9%) and college-educated (95.3% bachelor’s or graduate degree). They primarily identified as white (47.6%) or Asian (38.1%). Partner participation in Centering Pregnancy^®^ in-person meetings was similarly high, with 93.5% attending all or most sessions (not shown). Partners most frequently reported births occurring in 2020 (33.3%) or 2021 (19.0%).

## Participation in social media groups adjunct to centering pregnancy^®^

Most participants reported that their Centering Pregnancy^®^ groups engaged in communication outside of in-person meetings during pregnancy (79.1%) and postpartum (74.2%), with high levels of personal participation in these communications (80.0% and 76.4%, respectively; Table 2). WhatsApp was the most commonly used platform (62.9%), followed by email (43.6%), Slack (13.6%) and Facebook (12.1%). Social media groups often included both pregnant/birthing individuals and their partners (58.7%).

**Table 2:** Adjunct social media group participation among UCSF centering Pregnancy^®^ participants and partners engaging in social media survey

	Participant (*n* = 155)		Partner (*n* = 22)	
	*n*	%	*n*	%
Group communicated online				
No	8	5.2	1	4.5
During pregnancy	123	79.4	13	59.1
During postpartum	115	74.2	16	72.7
Don’t know	9	5.8	1	4.5
Participated in group’s online communications	(*n* = 140)		(*n* = 19)	
No^a^	7	5.0	8	42.1
During pregnancy	112	80.0	8	42.1
During postpartum	107	76.4	8	42.1
Platform used for communication	(*n* = 140)			
Email	61	43.6	4	21.1
Facebook	17	12.1	3	15.8
Slack	19	13.6	2	10.5
Zoom	12	8.6	1	5.3
WhatsApp	88	62.9	5	26.3
Google	2	1.4	0	0.0
Group target	(*n* = 143)			
All group participants	84	58.7	11	52.4
Pregnant people only	59	41.3	10	47.6
Proportion of group that participated in online group	(*n* = 142)			
All	23	16.2	1	5.3
Most	73	51.4	8	42.1
Some	36	25.4	7	36.8
Few	8	5.6	1	5.3
Do not remember	2	1.4	2	10.5
How often posted content during pregnancy	(*n* = 97)		(*n* = 7)	
Monthly	53	54.6	4	57.1
Weekly	21	21.6	3	42.9
Several times per week	22	22.7	0	0.0
Daily	1	1	0	0.0
How often read content during pregnancy	(*n* = 102)		(*n* = 8)	
Monthly	29	28.4	2	25.0
Weekly	24	23.5	2	25.0
Several times per week	26	25.5	1	12.5
Daily	15	14.7	3	37.5
Several times per day	8	7.8	0	0.0
How often posted content postpartum	(*n* = 101)		(*n* = 7)	
Monthly	46	45.5	3	42.9
Weekly	26	25.7	3	42.9
Several times per week	20	19.8	1	14.3
Daily	4	4	0	0.0
Several times a day	5	5		
How often read content postpartum	(*n* = 103)		(*n* = 7)	
Monthly	26	25.2	2	28.6
Weekly	17	16.5	3	42.9
Several times per week	28	27.2	2	28.6
Daily	17	16.5	0	0.0
Several times per day	15	14.6	0	0.0
How long after delivery continued to interact with group online	(*n* = 123)		(*n* = 14)	
Still meeting	24	19.5	0	0.0
4–6 weeks	18	14.6	5	35.7
6–12 weeks	6	4.9	1	7.1
3–6 months	6	4.9	1	7.1
6–12 months	21	17.1	1	7.1
12 + months	48	39	6	42.9
Reason participated in group				
Social support	121	78.1	8	36.4
Knowledge sharing	101	65.2	10	45.5
Trusted friends	27	17.4	5	22.7
Other	9	5.8	0	0.0
Would you have liked to have health provider join social media group			(*n* = 11)	
No	38	38.8	1	9.1
Yes	41	31.1	3	27.3
Unsure	53	40.2	7	63.6
Social media group still active			(*n* = 19)	
No	38	27.0	2	10.5
Yes	91	64.5	10	52.6
Don’t know	12	8.5	7	36.8
Participated in other online social media/in-person support groups				
No	41	26.5	17	77.3
Social media	92	59.4	4	18.2
In-person	41	26.5	0	0.0

^a^Reasons for non-participation in group’s online communications were reported for CP Participants as other (n = 4), not enough time (n = 2), not comfortable with social media (n = 1), not comfortable sharing (n = 1), and no access to platform (n = 1). Partners reported other (n = 5), did not need additional support (n = 2), did not need additional information (n = 2), not comfortable with social media (n = 1), not enough time (n = 1), and no access to platform (n = 1)

Participation in social media groups was robust, with two-thirds of participants reporting that all or most of their Centering Pregnancy^®^ group members were involved (67.6%). During pregnancy, most participants reported reading content frequently, with 48.0% engaging at least weekly. Posting content was less frequent, with 54.6% posting content monthly. Postpartum engagement was higher, with 54.5% of participants posting content at least weekly and 63.3% reading content at least several times per week. Participants primarily engaged in social media groups for social support (78.1%) and knowledge sharing (65.2%). Additionally, 59.4% of participants reported engaging in other online support groups while 26.5% participated in in-person support groups.

In contrast to birthing individuals, partners reported engagement in social media groups with 42.1% engaging during pregnancy and postpartum, primarily for knowledge sharing (45.5%), and social support (36.4%; Table 2). Most partners (77.3%) did not participate in other social media groups. Notably, birthing individuals reported higher partner engagement in in-person (78.6%) and social media groups (18.1% during pregnancy and 17.0% postpartum; not shown) than was self-reported by partners.

### In-person engagement with centering pregnancy^®^ group outside of scheduled group meetings

Unfacilitated in-person meetings among Centering Pregnancy^®^ group members were reported by 29.0% during pregnancy and 58.1% postpartum (Table 3). In-person meetings largely occurred every few months (60.0%) or monthly (11.1%) during pregnancy and every few months (45.6%), monthly (17.8%) or weekly (12.2%) postpartum. Many participants continued in-person interactions with their group for a year or more after giving birth (39.4%), with (20.2%) reporting ongoing meetings at the time of the survey. No relationship was identified between the frequency of posting or reading social media and in-person meeting attendance (not shown).

**Table 3:** In-person engagement with centering Pregnancy^®^ group among UCSF centering Pregnancy^®^ participants and partners engaging in social media survey

	Participant (*n* = 155)		Partner (*n* = 22)	
	*n*	%	*n*	%
Group met in person outside of regular sessions	(*n* = 155)			
No	57	36.8	9	40.9
Yes, in pregnancy	45	29.0	4	18.2
Yes, postpartum	90	58.1	12	54.6
Frequency of in-person meetings during pregnancy	(*n* = 45)		(*N* = 4)	
Every few months	27	60.0	0	0.0
Monthly	5	11.1	3	75.0
Weekly	3	6.7	0	0.0
Several times per week	1	2.2		
Other	9	20.0	1	25.0
Frequency of in-person meetings postpartum	(*n* = 90)		(*n* = 12)	
Every few months	41	45.6	6	50.0
Monthly	16	17.8	3	25.0
Weekly	11	12.2	0	0.0
Several times a week	2	2.2	0	0.0
Other	20	22.2	3	25.0
How long after delivery continued to interact with group in-person	(*n* = 104)		(*n* = 12)	
Still meeting	21	20.2	0	0.0
4–6 weeks	15	14.4	2	16.7
6–12 weeks	6	5.8	1	8.3
3–6 months	8	7.7	2	16.7
6–12 months	13	12.5	1	8.9
12 + months	41	39.4	6	50.0

###  Impact of COVID-19 on centering pregnancy^®^ social media group

The COVID-19 pandemic influenced social media group dynamics among some Centering Pregnancy^®^ participants. Among participants with ongoing groups, 41.9% reported no change in interactions, while 24.7% reported more frequent interactions, and 33.3% reported less frequent interactions (not shown). One-third of ongoing group participants reported changes in group interaction content due to COVID-19 (34.8%), with discussions shifting to pandemic-related stress, mental health, managing risks, working from home, childcare, and ideas for home-based activities. Several participants also shared that meet-ups occurred less frequently or ceased altogether due to social distancing, and some members moved away. Representative participant narratives highlighted these shifts:

*We talk about how to manage risks for our families*,* the decision to go back to daycare*,* etc. many have also since moved away*,* so the WhatsApp group has taken on new significance in that it’s really the only contact we’ll still have with each other on a regular basis. Before Covid we’d get together almost monthly with the babies.*

*Now that people are varied in the activities they engage in*,* some are more hunkered down than others*,* there isn’t as much conversation. Back when we were first newborn moms*,* we were on a journey together and could also get together more frequently*,* so we would be chatting each other at all hours of the day for advice*,* feedback or just venting.*

*All of us delivered shortly before social distancing began*,* so we were never able to meet up in person postpartum. We spoke more about navigating postpartum without additional help from family*, etc.

### Topics most discussed and additional information desired

The most commonly discussed topics among birthing individuals included birth experience, general pregnancy questions, common discomforts of pregnancy, and infant sleep (mentioned by 40% or more; Table 4). Partners listed fewer discussion topics than birthing individuals, focusing primarily on products and services, pregnancy questions, infant feeding, sleep, child development, and introducing solids foods.

**Table 4:** Topics discussed and discussion needs during adjunct social media group participation for UCSF centering Pregnancy^®^ participants and partners engaging in social media survey

	Discussed		Wanted more discussion	
	*n*	%	*n*	%
***Centering Pregnancy***^®^ ***Participants (N = 155)***				
Birth experience	104	67.1	32	20.6
General pregnancy questions	96	61.9	11	7.1
Common discomforts of pregnancy	84	54.2	16	10.3
Infant sleep	66	42.6	66	42.6
Maternal recovery/health	60	38.7	60	38.7
Pregnancy complications	59	38.1	24	15.5
Breastfeeding/formula feeding	58	37.4	58	37.4
Newborn health and care (bathing, skin care, colic, allergies, vaccinations)	53	34.2	53	34.2
Child development	42	27.1	42	27.1
Maternal mood	35	22.6	35	22.6
Introduction of solid food	32	20.6	32	20.6
Partner relationship	31	20	31	20.0
Return to sex, family planning, sex drive	29	18.7	29	18.7
Products and services	23	14.8	23	14.8
Other	8	5.2	8	5.2
***Partners (N = 22)***				
Products and services	10	45.5	0	0.0
General pregnancy questions	9	40.9	2	9.1
Breastfeeding/formula feeding	9	40.9	1	4.5
Infant sleep	9	40.9	4	18.2
Child development	9	40.9	2	9.1
Introduction of solid food	9	40.9	2	9.1
Birth experience	8	36.4	3	13.6
Maternal recovery/health	8	36.4	2	9.1
Newborn health and care (bathing, skin care, colic, allergies, vaccinations)	8	36.4	2	9.1
Common discomforts of pregnancy	7	31.8	1	4.5
Pregnancy complications	7	31.8	0	0.0
Maternal mood	5	22.7	1	4.5
Partner relationship	3	13.6	3	13.6
Return to sex, family planning, sex drive	2	9.1	1	4.5
Other	2	9.1	0	0.0

Newborn health and care^a^ (bathing, skin care, colic, allergies, vaccinations)

The most frequently reported topics birthing individuals wanted more information on were infant sleep (42.6%), maternal recovery/health (38.7%), breastfeeding/formula feeding (37.4%), newborn health and care (34.2%), and child development (27.1%; Table 4). Partners expressed limited interests, mainly in sleep (18.2%), birth experience (13.6%) and partner relationships (13.6%).

### Emotional and informational support needs and mental health during pregnancy and postpartum

Most birthing individuals reported that their emotional (86.5%) and informational (91.0%) support needs were very or somewhat well met during pregnancy, but these proportions were lower postpartum (63.3% for emotional and 69.7% for informational; Table 5). Depressive and anxiety symptoms was significantly higher postpartum compared to during pregnancy, with 5.8% reporting depressive symptoms always or often antenatally compared to 18.6% postpartum (*p* < 0.002), and 9.0% reporting anxiety symptoms always or often antenatally compared to 22.1% postpartum (*P* = 0.009).

**Table 5:** Emotional and informational support needs and depressive and anxiety symptoms, during pregnancy and postpartum among UCSF centering Pregnancy^®^ participants and partners engaging in social media survey

	Centering Participant (*n* = 155)				Partner (*n* = 12)			
	Pregnancy		Postpartum		Pregnancy		Postpartum	
	n	%	n	%	n	%	n	%
How well were your emotional support needs met?			(*n* = 142)					
Very well	70	45.2	35	24.6	6	28.6	5	26.3
Somewhat well	64	41.3	55	38.7	7	33.3	4	21.1
Neutral	13	8.4	23	16.2	7	33.3	5	26.3
Not very well	5	3.2	21	14.8	0	0.0	4	21.1
Poorly	3	1.9	8	5.6	1	4.8	1	5.3
How well were your informational support needs met?			(*n* = 142)					
Very well	92	59.4	46	32.4	6	28.6	2	10.5
Somewhat well	49	31.6	53	37.3	11	52.4	10	52.6
Neutral	7	4.5	20	14.1	4	19.0	6	31.6
Not very well	6	3.9	19	13.4	0	0.0	1	5.3
Poorly	1	0.6	4	2.8	0	0.0	0	0.0
Frequency of depressive symptoms			(*n* = 140)					
Always	0	0.0	4	2.9	0	0.0	0	0.0
Often	9	5.8	22	15.7	2	9.5	3	15.8
Sometimes	32	20.6	48	34.3	6	28.6	5	26.3
Rarely	65	41.9	52	37.1	10	47.6	9	47.4
Never	49	31.6	14	10	3	14.3	2	10.5
Frequency of anxiety symptoms	(*n* = 154)		(*n* = 140)					
Always	1	0.6	7	5	0	0.0	0	0.0
Often	13	8.4	24	17.1	4	19.0	5	26.3
Sometimes	39	25.3	38	27.1	4	19.0	3	15.8
Rarely	54	35.1	51	36.4	6	28.6	7	36.8
Never	47	30.5	20	14.3	7	33.3	4	21.1
Mental health diagnoses								
Depression	9	6.2	17	11.3	0	0.0	1	4.8
Anxiety	12	7.7	30	19.4	0	0.0	2	9.1

Higher frequency of social media group engagement was associated with greater odds of depressive symptoms during pregnancy and greater odds of anxiety symptoms postpartum (Table 6). Specifically, each higher category of engagement frequency in reading group content was associated with a 2.32-fold (95% CI 1.17–4.61) increased odds of reporting depressive symptoms always or often. Each higher category in engagement frequency in reading group content was associated with a 1.60-fold increased odds of reporting anxiety always or often postpartum (95% CI 1.10–2.34) while increased posting of content was associated with a 1.51-fold increased odds of postpartum anxiety (95% CI 1.01–2.27).

**Table 6:** Emotional and informational support need*s and* depressive and anxiety symptoms, during pregnancy and postpartum among UCSF centering Pregnancy^®^ participants

	Pregnancy				Postpartum			
	Depressive symptoms		Anxiety		Depressive symptoms		Anxiety	
	OR (95% CI)	*P*	OR (95% CI)	*P*	OR (95% CI)	*P*	OR (95% CI)	*P*
Frequency *read* social media group content	2.32 (1.17–4.61)	0.016	1.09 (0.62–1.92)	0.77	1.29 (0.89–1.86)	0.181	1.60 (1.10–2.34)	0.015
Frequency *posted* social media group content	1.51 (0.65–3.52)	0.342	0.72 (0.28–1.85)	0.49	1.03 (0.66–1.60)	0.897	1.51 (1.01–2.27)	0.046

Depressive symptoms and anxiety operationalized as always or often versus sometimes, rarely or never (reference)

Partners reported lower levels of emotional and information support, with unmet needs more pronounced postpartum (Table 5). Emotional support decreased from 61.9% to 47.4% postpartum, and informational support from 81.0% to 63.1%. Depressive symptoms increased from 9.5% during pregnancy to 15.8% postpartum, and anxiety symptoms from 19.0% to 27.3%, though differences were not statistically significant.

## Discussion

### Key findings and implications

This study highlights the heightened need for emotional and informational support and increasing participation in social media groups among both birthing individuals and their partners during the postpartum period. Social media group engagement was valued by participants, as others have reported, [8, 26] and served as an important platform for discussing diverse topics in maternal and infant care and connecting with parents of similarly-aged infants. Yet participants expressed a desire for more information on certain topics, particularly infant feeding, sleep and other care and maternal recovery. These findings suggest a clear opportunity to enhance postpartum support through interventions such as resource packages accessible via social media, facilitated postpartum support groups, and in-person postpartum support sessions. More broadly, these findings underscore the need to address the postpartum period, a time often neglected by traditional healthcare models, by providing additional and alternative avenues for information and social support. Peer-learning group models offer a cost-effective and efficient opportunity for meeting these perinatal support needs.

### Partner engagement and support

Though the sample of partners was small, limiting generalizability, the findings offer valuable insights into partner engagement and support [27]. Partners reported greater interest and activity in social media groups during the postpartum period, with many engaging to connect with “trusted friends”. Unlike birthing individuals, they were less involved in other social media groups, presenting an interesting opportunity for targeted interventions to better engage and support this population for their own wellbeing as well as supporting their ability to support their birthing partners, optimizing their wellbeing [28]. Prior research has shown that partners desired guidance and social support in the postpartum period for navigating infant care and partner relationship changes, [29] as well as around postpartum depression, which can afflict both birthing individuals and partners [27, 30]. In our small sample, compared to birthing individuals, partners reported greater unmet emotional support needs during both pregnancy and postpartum, greater frequency of depressive and anxiety symptoms during pregnancy, and similar levels of symptoms postpartum, highlighting important gaps. Research suggests an important gap in the evidence on digital interventions targeting fathers during the perinatal period [31]. By considering the unique preferences and challenges experienced by partners of birthing individuals during pregnancy and postpartum, [32] intervention models could improve engagement, reduce barriers to support, and address critical gaps in partner mental health [29]. Strategies could include tailored approaches, such as partner-specific content, partner-focused peer groups, or moderated forums, to meet the educational and support needs of this population.

### Facilitation of social media groups

Participants expressed mixed opinions regarding healthcare provider facilitation of social media groups, with responses divided between a preference for facilitation, no facilitation, and uncertainty. Unfacilitated, peer-led models are low-cost and scalable, making them easier to implement compared to provider-facilitated models, which require more resources but ensure greater control over the quality of information shared. Previous intervention studies focused on postpartum depression have demonstrated similar outcomes for both models, [33, 34] suggesting that either approach can be effective depending on participant preferences. Program developers may consider offering hybrid models that combine facilitation with independent social media participation or providing participants with options for facilitation levels based on their needs.

### Impact of COVID-19 pandemic

Although conceptualized before the COVID-19 pandemic, this study provides timely insights into the role of social media support during a period marked by increased virtual care delivery. Social media groups were highly valued during the pandemic, not only for disseminating information but also for fostering social support in a time of heightened mental health challenges and limited in-person interaction. Our findings echo recent studies showing that the pandemic contributed to adverse postpartum mental health outcomes, with loneliness identified as a key contributing factor [35]. Social media interventions can serve as effective tools for addressing connection, both during and beyond the COVID-19 pandemic.

### Strengths and limitations

This study has several strengths. It investigates a new practice among postpartum individuals, a life stage traditionally underserved by health care systems, during a period when mobile and online resources are increasingly important. It also captures unique perspectives on social media use during the COVID-19 pandemic. However, it did have several limitations. Recruitment was relatively low and selective due to reliance on UCSF’s MyChart portal messages, which many eligible participants never accessed. Consequently, participants may skew toward individuals who are more actively engaged in health communication and provider connection via this portal. Partner recruitment was also limited as it relied on birthing individuals to forward the recruitment link. Reasons for low partner participation remain unclear. Additionally, the sample reflects the UCSF Centering Pregnancy^®^ population, which is more educated, mostly white, and largely compose of first-time parents, limiting the generalizability of our findings within the broader US birthing population. Mental measures of mental health symptoms were limited by the need to reduce participant burden among our new parent population, some of our measures were limited (e.g., report of depression and anxiety symptoms). Finally, the pandemic timing and online nature of data collection may have influenced responses or perceptions social media/online interactions.

### Implications for practice and future research

The demonstrated interest in postpartum online support groups suggests that providers should consider recommending patient-organized, unfacilitated social media support groups as a cost-effective and accessible resource. Providers could also maintain curated lists of facilitated social media support groups tailored to specific populations, issues, or local communities to offer their patients additional support options.

Our study’s examination of key discussion topics and areas where additional discussion or information is needed underscores critical opportunities for advancing educational resources to support this population. The findings revealed notable differences in unmet informational needs between birthing individuals and their partners, which have important implications for support structures and content. Specifically, birthing individuals reported a broader range of unmet informational needs, seeking information on topics such as infant sleep, maternal recovery and health, breastfeeding versus formula feeding, newborn care and health, and child development. In contrast, partners tended to focus on fewer topics, with infant sleep, the birth experience, and partner relationships being prioritized. This divergence highlights the need for tailored programming for both groups. Birthing individuals may benefit from diverse, detailed, and personalized informational resources, while partners may require more targeted support that emphasizes their roles and interactions with the birthing individual and child. The extent to which partner’s lower engagement in social media groups and adjunct resources, compared to birthing individuals, contributes to these differences in unmet informational needs is unclear.

The association between frequent social media support group use and depressive and anxiety symptoms merits further investigation. This correlation has been identified in a few other studies, [36, 37] and future research should seek to delineate directionality of this relationship, i.e., whether social media use during pregnancy and postpartum negatively impacts mood through mechanisms such as social comparison [38] or whether individuals experiencing depression or anxiety are more likely to engage in social media groups in search of informational or emotional support. A clear understanding of risk-benefit dynamics is critical for designing effective online support programs as social media continues to play a central role in communication and education worldwide.

### Conclusions and next steps

Social media interventions may offer a promising avenue for addressing unmet postpartum support needs for birthing individuals and their partners in the evolving digital health landscape. Leveraging social media within the post-COVID Centering Pregnancy^®^ model offers an opportunity to extend support into the postpartum period, potentially enhancing program reach while maintaining efficiency. Careful consideration must be given to balancing model benefits with potential risks, particularly as it relates to ensuring information is evidence-based and addressing mental health concerns. Next steps should include co-designing social media-based support platforms in collaboration with patients to ensure these interventions are patient-centered and responsive to diverse needs. Piloting moderated postpartum forums could serve as an initial step in evaluating the feasibility, effectiveness, and safety of these approaches. Further research should also explore optimal strategies for improving partner engagement, addressing preferences for facilitated versus unfacilitated groups, and refining the integration of social media support within peer-learning and support models.

## Data Availability

Data are accessible upon reasonable request by the corresponding author.
